# The Roles of Acidosis in Osteoclast Biology

**DOI:** 10.3389/fphys.2016.00222

**Published:** 2016-06-24

**Authors:** Feng-Lai Yuan, Ming-Hui Xu, Xia Li, He Xinlong, Wei Fang, Jian Dong

**Affiliations:** ^1^Department of Orthopaedics and Central Laboratory, The Third Hospital Affiliated to Nantong UniversityWuxi, China; ^2^Department of Neurosurgery, Wuxi Ninth People's Hospital Affiliated to Soochow UniversityLiangxi Road Wuxi, China; ^3^Department of Orthopedic Surgery, Zhongshan Hospital, Fudan UniversityShanghai, China

**Keywords:** acidosis, osteoclast, osteoclast activity, osteoclast survival, osteoclast adhesion

## Abstract

The adverse effect of acidosis on the skeletal system has been recognized for almost a century. Although the underlying mechanism has not been fully elucidated, it appears that acidosis acts as a general stimulator of osteoclasts derived from bone marrow precursors cells and enhances osteoclastic resorption. Prior work suggests that acidosis plays a significant role in osteoclasts formation and activation *via* up-regulating various genes responsible for its adhesion, migration, survival and bone matrix degradation. Understanding the role of acidosis in osteoclast biology may lead to development of novel therapeutic approaches for the treatment of diseases related to low bone mass. In this review, we aim to discuss the recent investigations into the effects of acidosis in osteoclast biology and the acid-sensing molecular mechanism.

## Introduction

The maintenance of intracellular and extracellular physiological pH is crucial for normal cell function. A shift in pH toward a more acidic environment can lead to: (a) systemic acidosis, which develops due to pathological conditions like renal and respiratory diseases, diabetes, anemia, menopause, and aging and leads to abnormal cell function; and (b) localized extracellular acidosis, which results from both pathological and physiological conditions, such as ligand–receptor interactions in the tumor microenvironment, infection, inflammation, and wound healing (Arnett, 2008, 2010; Ahn et al., 2012). Localized acidosis appears to play a role in early wound healing, infectious diseases, tumorigenesis, and bone remodeling (Belinsky and Tashjian, 2000); however, the mechanism underlying localized acidosis is poorly understood. Typically, extracellular acidosis promotes inflammatory cell defense against pathogens by regulating migration and phagocytosis (Martinez et al., 2006). However, lactic acidosis caused by increase in glycolysis during tumorigenesis facilitates tumor invasion and metastasis, and has deleterious effects on biological processes (Rofstad et al., 2006; Sharma et al., 2015). Recent results from *in vivo* studies have shown that bone loss related to acidosis was not due to passive physicochemical dissolution of bone minerals but rather increased osteoclastic resorption (Kraut et al., 1984; Meghji et al., 2001).

Osteoclasts are the only cells known to be involved in resorption of large quantities of bone material and mineralized cartilage (Song et al., 2009; Li et al., 2010; Liu et al., 2015). Osteoclasts are large multinucleated cells generated by the fusion of mononuclear precursor cells derived from hematopoietic stem cells in response to specific molecular signals (Kelemen, 1986). The process of osteoclast differentiation is mainly regulated by two cytokines, namely, macrophage colony-stimulating factor (M-CSF) and receptor activator of nuclear factor-kappa B (NF-κB) ligand (RANKL) (Boyle et al., 2003; Wada et al., 2006). M-CSF has been reported to play a crucial role in the proliferation and survival of osteoclast precursor cells (Hamilton and Anderson, 2004). Moreover, other studies suggest that osteoclasts can also be regulated by several hormones, including parathyroid hormone (Furuya et al., 2011), glucocorticoids (Fujihara et al., 2014), 1,25(OH)2D3 (Geusens et al., 1991), and estrogen (Piao et al., 2016), by influencing their formation, bone resorption activity and life span, by regulating expression of various molecular factors. In addition, multiple studies have identified that acidic extracellular pH can considerably induce bone loss through increasing osteoclast differentiation, survival, and activity. This is likely due to the increased expression of osteoclastogenic and bone-resorptive regulatory molecules, such as transcription factor NFATc1 (Komarova et al., 2005; Pereverzev et al., 2008; Li et al., 2013; Yuan et al., 2014), cathepsin K (Muzylak et al., 2007), carbonic anydrase II (Biskobing and Fan, 2000), the vacuolar-type H+-ATPase (Nordstrom et al., 1997; Kim et al., 2007), and osteopontin (Kim et al., 2007).

This review focuses on recent investigations into the effects of acidosis on osteoclasts. Additionally, we have discussed the molecular role of acid/proton sensing genes in the regulation of osteoclast differentiation, survival, and activity, with an aim to understand the underlying mechanisms of acidosis-induced osteoclastic bone resorption.

## Functions of acidosis in osteoclast biology

### Fusion and differentiation of preosteoclast cells

Osteoclast differentiation involves 3 general steps: (a) differentiation of monocytic/macrophage lineage precursor cells into preosteoclast cells expressing mononuclear tartrate-resistant acid phosphatase (TRAP) gene (TRAP^+^), (b) Fusion of these TRAP-positive mononuclear preosteoclasts to become non-functional polykaryons, and finally, (c) the activation of these nonfunctional multinucleated cells into fully activated functional osteoclasts (Shiotani et al., 2002; Nakamura et al., 2007). Osteoclast differentiation is regulated by two key growth factors, M-CSF and RANKL (Feng et al., 2014). M-CSF, c-Fms is critical for survival and differentiation of osteoclast precursors into preosteoclasts (Hamilton and Anderson, 2004), and RANKL is involved in eliciting different cellular responses, including preosteoclast fusion, polykaryon formation, and osteoclasts activation (Asagiri and Takayanagi, 2007). In addition, RANKL also activates nuclear factor-activated T cells c1 (NFATc1), responsible for preosteoclast fusion and differentiation (Takayanagi, 2007; Kajiya, 2012).

Recently, it has been reported that acidosis-mediated induction of osteoclast formation is most effective in the later stages of preosteoclast differentiation (Kato and Morita, 2011; Kato and Matsushita, 2014). To identify the stage at which acidosis promotes osteoclast differentiation, bone marrow cell cultures were maintained in medium, containing RANKL and M-CSF cytokines, at pH 7.4 for the first 3 days, followed by replacing the medium with extracellular acidic pH of 6.8 for 1 day during the 4-day culture period (Kato and Morita, 2011). This led to marked increase in the number of osteoclasts. However, cultures kept in medium with pH 7.4 for 4 days rarely induced large positive multinuclear osteoclasts. These findings indicated that acidosis promotes osteoclast differentiation by targeting the later stage of preosteoclast differentiation. Moreover, it was also observed that bone marrow cells stimulated with acidosis for more than 6 h after 3-day culture at pH 7.4 had increased osteoclast size.

### Osteoclast activity

A variety of factors, including cytokines and immune complexes, lead to maturation and activation of multinucleated osteoclasts that subsequently initiate bone remodeling. Interestingly, accumulating evidence has indicated that osteoclasts are very sensitive to small changes in pH and become activated by acidosis (Krieger et al., 1992). Osteoclast activity induced by acidosis was described by Teti et al. (1989), who reported that extracellular acidification of osteoclasts reduced cytosolic calcium, and accelerated the expression of cell-matrix adhesion proteins. However, few earlier studies identified that acid ingestion leads to reduction in bone mass and increase in osteoclastic resorption surface (Barzel and Jowsey, 1969; Chan et al., 1985; Arnett and Dempster, 1986).

The attachment structures (i.e., podosomes) are located in areas where osteoclasts adhere to bone during osteoclastic resorption (Teti et al., 1989). Meghji et al. (2001) reported that acidosis induced extensive osteoclastic resorption cavities as evident from the scalloped edges in 3-day cultures of neonatal mouse calvaria. Occasionally, acidosis also induced aggressive osteoclastic resorption resulting in complete bone perforation. However, the treatment of cultured neonatal mouse calvariaes with alkalotic medium decreased the osteoclastic activity and increased osteoblastic formation (Bushinsky, 1996). Another study showed that magnesium hydroxide temporarily increased osteoblast activity and suppressed osteoclast number in peri-implant bone remodeling (Janning et al., 2010). These findings clearly indicated that modulation of osteoclast activity is determined by acidosis and dependent on pH in mouse calvarial cultures. Acidosis-induced resorptive activity of rat osteoclasts was similar to as observed *in vivo*. Thus, these experiments confirmed that osteoclasts have little or no resorptive activity in the long bones of neonatal rats when ambient pH was above 7.3, but it increased steeply with a fall in the pH and reached its maximum level at pH 6.8. Extracellular H^+^ led to pH reduction of less than 0.1 unit, but bone resorption was increased 2-fold, suggesting an important contribution of acidosis in promoting osteoclast activity (Arnett and Spowage, 1996). Moreover, *in vitro* cell culture experiments suggested that acidosis led to a direct stimulatory effect on bone resorption by osteoclasts cultured on bone slices (Arnett and Dempster, 1986; Arnett, 2010). Similarly, extracellular acidosis also induced osteoclast activity in avian and human osteoclasts (Arnett and Dempster, 1987; Arnett, 2008). In parallel, direct stimulation of osteoclasts by acidosis also induced rapid and significant increase in intracellular Ca^2+^ ([Ca^2+^]i) concentration, which further stimulated the nuclear translocation/activation of NFATc1 and promoted osteoclasts bone resorption activity (Komarova et al., 2005).

Acidosis seems to induce osteoclast activity by both direct and indirect effects on osteoblasts. Several factors expressed by osteoblasts might regulate osteoclast activity. Acidosis-stimulated production of prostaglandin E2 from osteoblasts increased osteoclastic bone resorption (Krieger et al., 2000; Bushinsky et al., 2001; Frick and Bushinsky, 2003). Other factors secreted by osteoblasts, such as RANKL, M-CSF, and osteoprotegerin (OPG), have also been known to modulate osteoclast precursor differentiation and osteoclast activity. RANKL expressed at the cell surface of osteoblasts interacts with its receptor RANK and induce the resorptive activity of osteoclasts (Lacey et al., 1998; Tsurukai et al., 2000). Metabolic acidosis also increases the osteoblast autocrine or paracrine production of RANKL. This up-regulation of RANKL expression under acidic conditions depends on cyclo-oxygenase activity, which likely stimulates osteoclast activity (Frick and Bushinsky, 2003).

### Osteoclast survival, adhesion, and migration

Osteoclasts are multinucleated and terminally differentiated cells with a short life span (Miyazaki et al., 2000) and apoptosis has been identified as the major form of cell death, both *in vitro* and *in vivo* (Xing and Boyce, 2005; Mollazadeh et al., 2015). Interestingly, extracellular acidosis and RANKL have not only been identified as potent stimulators of osteoclast resorptive activity and differentiation, but also inhibit osteoclast apoptosis, contributing to the enhancement of osteoclast life span. Moreover, it has also been reported that acidosis exerts its direct effect on osteoclast survival through the activation of NFATc1. Several other pathways have also been suggested to contribute into the effects of acidosis on osteoclast apoptosis and survival. For example, suppression of cytosolic free calcium concentration ([Ca^2+^]i) using the intracellular Ca^2+^ chelator, BAPTA, abolished the ability of acidosis to increase osteoclast survival. Calcium signaling results in activation of protein kinase C (PKC), which either regulates the phosphorylation status of pro- or anti-apoptotic proteins, or promotes ERK1/2 phosphorylation. Activation of the MAPK pathway is known to be important for osteoclast survival. Studies using PKC inhibitor also showed that pharmacological inhibition of PKC completely blocked acidosis-induced prolongation of osteoclasts survival, suggesting that acidification-increased osteoclast lifespan was dependent on PKC activation. Although activation and nuclear translocation of NFATc1 have been suggested to be critical for osteoclast differentiation and activity under acidic conditions (Komarova et al., 2005), studies using NFAT-specific inhibitor, 11R-VIVIT, showed that NFATc1 inhibition had no effect on acid-induced prolongation of osteoclast survival.

Osteoclastic bone resorption by mature osteoclasts involves multiple steps: (1) fusion of mononuclear pre-fusion osteoclasts into multinucleated osteoclasts; (2) attachment of osteoclasts to the bone surface; (3) polarization [characterized by ruffled border and clear zone (actin ring)], increased secretion of acid and lysosomal enzymes into the space beneath the ruffled border; and (4) apoptosis (Suda et al., 1997). Ahn *et al*. demonstrated that acidosis promoted osteoclast formation and function through increased adhesion and migration (Ahn et al., 2012). To further support the role of acidosis in mediating osteoclast survival, and to evaluate whether osteoclast spreading, adhesion, and migration played a key role in determining bone-resorptive osteoclast function, RANKL induced osteoclasts were exposed to HEPES-buffered media at pH 7.0 or 7.5 (Ahn et al., 2012). These extracellular acidification experiments demonstrated that osteoclasts containing more than 3 or 10 nuclei and apparent actin rings survived longer at a relatively low pH. Moreover, osteoclasts cultured under the same condition exhibited increased osteoclast adhesion and migration at pH 7.0 as compared to pH 7.5, but failed to spread. These observations indicated that acidosis plays a critical role in osteoclast survival, adhesion, and migration.

Integrins are cellular adhesion receptors that belong to a superfamily of receptors that are involved in mediating cell–matrix interactions. Osteoclasts typically exhibit high expression of transmembrane integrin-αvβ3 heterodimer, which recognize Arg-Gly-Asp (RGD) motif on the bone matrix components, osteopontin and bone sialic protein (Mchugh et al., 2000; Rao et al., 2006). The inhibition of αvβ3 integrin was shown to suppress *in vitro* and *in vivo* bone-resorption activity, suggesting that it might play a major role in regulating osteoclasts function (Novack and Faccio, 2011). In the study by Ahn et al. (2012), osteoclasts exposed to low pH led to increased secretion of osteopontin into the extracellular space of mature osteoclasts, while RGD peptide treatment that antagonize the matrix proteins, resulted in inhibition of acidosis-induced osteoclast adhesion and migration. Additionally, this study identified that extracellular acidosis also activated the osteoclast adhesive and migratory signal molecules independent of the αvβ3 integrin pathway, including Pyk2, Cbl-b, and Src signals. This observation again emphasized that extracellular acidosis increased bone resorption by enhancing osteoclast survival, adhesion, and migration.

## Mechanisms of acidosis sensing and its regulation in osteoclasts

Several types of plasma membrane sensors have been reported to be involved in osteoclasts acid-sensing, namely G-protein coupled receptors (GPCRs) and non-GPCR sensors (Damaghi et al., 2013). GPCRs include ovarian cancer G protein-coupled receptor 1 (OGR1) and T cell death-associated gene 8 (TDAG8), which have also been identified as acid receptors on osteoclasts (Yang et al., 2006; Pereverzev et al., 2008; Li et al., 2009; Hikiji et al., 2014). A key breakthrough in the field of proton-sensing mechanisms was the discovery of the proton-sensing ability of transient receptor potential (TRP) V1 (TRPV1) protein (Kajihara et al., 2010). This discovery suggested that non-GPCR proteins can also sense and respond to different extracellular pH environments (Kajihara et al., 2010). The acid-sensitive ion channel (ASIC) is a member of the non-GPCR protein family that has been shown to be expressed on human and rat osteoclasts and might have role in acidosis sensing (Jahr et al., 2005; Li et al., 2013). ASIC encodes at least six different subunits, including ASIC1a, ASIC1b, ASIC2a, ASIC2b, ASIC3, and ASIC4 (Waldmann et al., 1997; Li et al., 2014). Understanding of the functional molecular mechanism of these acid sensing sensors is likely to provide insights into their regulatory role under acidosis and may help to understand their link in modulating osteoclast biological behavior.

### Proton-sensing GPCRs

OGR1 was originally described as a receptor for sphingosylphosphorylcholine (Xu et al., 2000; Damaghi et al., 2013; Justus et al., 2013; Thongon et al., 2014), but later shown to be acting as a proton sensing receptor that couples with Gq protein (Ludwig et al., 2003). OGR1 was found to be expressed on osteoclasts like cells differentiated from RAW264.7 cells (Yang et al., 2006). OGR1 expression was also observed on the osteoclasts in bone marrow macrophages (BMMs) treated with RANKL to induce osteoclast differentiation (Yang et al., 2006). These studies suggested that OGR1 may act as a proton sensing receptor in osteoclasts.

**Figure 1:**
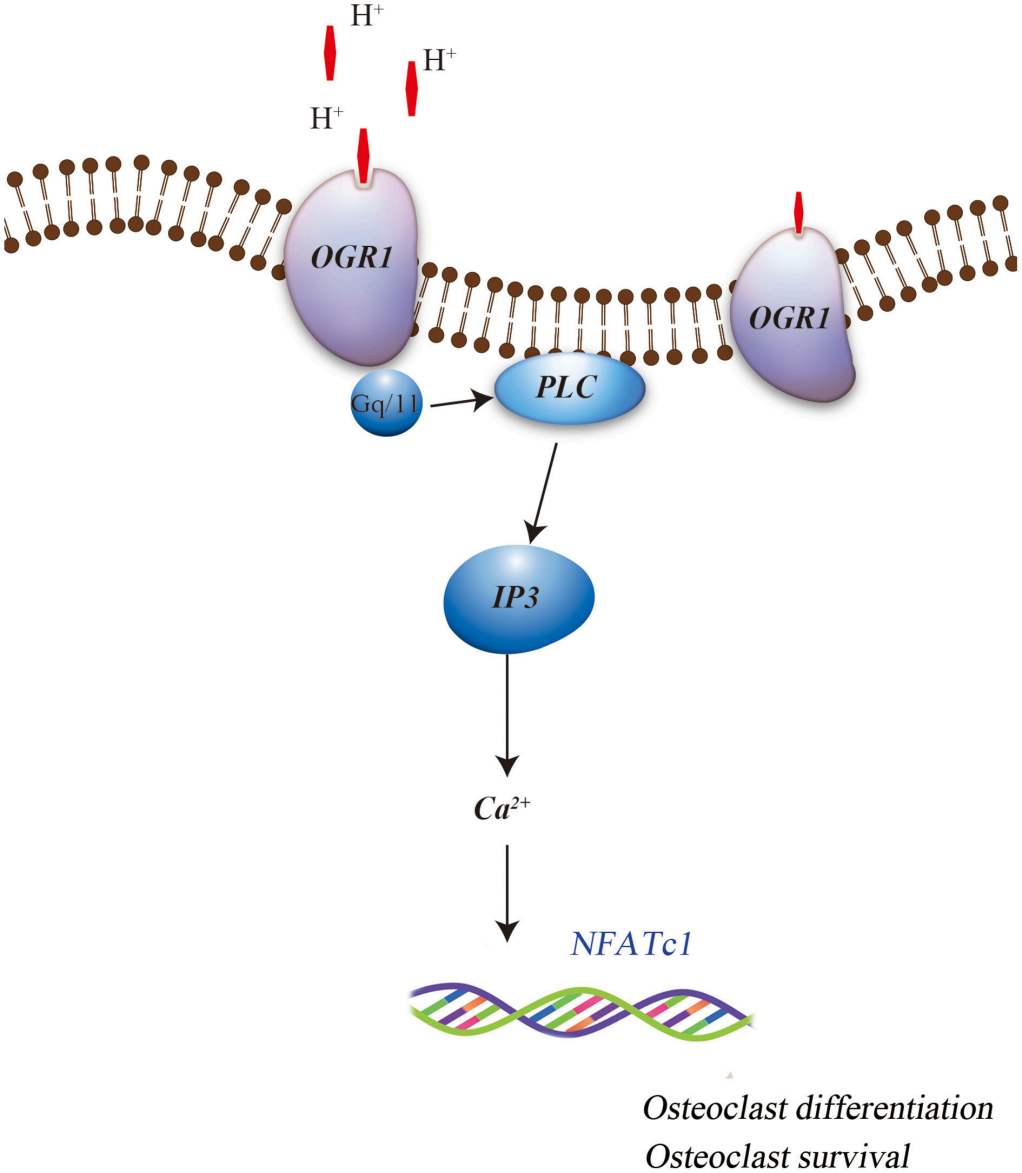
**Role of OGR1 in osteoclasts associated differentiation and survival**.

More specifically, OGR1 has been shown to be activated by acidosis to increase [Ca^2+^]i levels *via* Gq stimulation, which in turn resulted in cyclooxygenase 2 (COX-2) gene expression and subsequent prostaglandin E2 (PGE2) production. This regulation was sensitive to Gq/11 inhibitor and OGR1-specific siRNA, which stimulate bone resorption activity of osteoclasts by inducing the RANKL/RANK system. It has also been reported that extracellular acidic pH, mimics RANKL and induces the Ca^2+^/calcineurin/NFATc1 pathway, which is critical for osteoclast differentiation and function, possibly by activated OGR1 in the osteoclasts (Komarova et al., 2005; Pereverzev et al., 2008). In addition, Yang et al. (2006) have reported that OGR1 is expressed early during osteoclastogenesis, both *in vivo* and *in vitro*, and is crucial for osteoclast differentiation. Iwai et al. (2007) have identified RANKL-induced osteoclastogenesis by inhibiting the expression of regulator of G protein signaling 18 (RGS18), a negative regulator of the OGR1/NFATc1 pathway. This indicated the role of acid sensing OGR1 in osteoclasts differentiation and function. Recent findings have also suggested that OGR1 is involved in the acidosis-induced increase in osteoclast [Ca^2+^]i levels (Pereverzev et al., 2008). Interestingly, OGR1-mediated regulation of calcium signaling pathway during extracellular acidosis results in acidosis-induced osteoclast survival. OGR1 activation in osteoclast promoted survival by inducing the activation of protein kinase C (PKC) (Pereverzev et al., 2008). Frick et al. (2009) had shown that a non-specific inhibitor of OGR1 attenuated acidic pH-induced [Ca^2+^]i levels in primary calvarial cells and Ca^2+^ efflux from calvariae (Figure 1). This data suggests that acidic pH induced osteoclasts differentiation, function, and survival and may involve OGR1/Ca^2+^ signaling pathway. Further evidence for a positive role of OGR1 in osteoclastogenesis was detected in OGR1 knockout mice (Li et al., 2009; Okajima, 2013), where these mice showed a pH-dependent osteoclasts survival effect. However, *in vivo* X-ray scans did not show overall abnormality in the bones of these animals (Li et al., 2009). In addition, a recent publication further confirmed that *in vivo* loss of OGR1 increased bone mineral density probably by increasing the bone formation and directly contributing to the decreased bone resorption that was observed in rapidly growing mice (Krieger et al., 2016).

### TRP family

Among the TRP family members, TRPV1 is a calcium-permeable channel that has been widely studied and is activated only by severe acidosis resulting from pH values below 6 (Tominaga et al., 1998; Morales-Lazaro et al., 2013). Among the several recent reports suggesting the involvement of TRPV1 in osteoclast differentiation (Idris et al., 2010; Kato and Morita, 2013; Rossi et al., 2014a,b), TRPV1 specific agonist capsaicin, in particular, increased the osteoclasts formation in BMMs cultures treated with RANKL and M-CSF (Rossi et al., 2009). In contrast, the TRPV1 antagonist, capsazepine, resulted in inhibition of the osteoclast formation and their bone resorptive activity (Idris et al., 2010). It was also observed that TRPV1 blockade protected against ovariectomy induced bone loss in mice. However, Kato and Morita (2013) have recently reported that TRPV1 specific agonist, capsaicin, and its antagonist, AMG9810, neither promoted nor inhibited osteoclasts formation, respectively.

Likewise, another member of the TRP family, TRPV4, has also recently been reported to play a crucial role in osteoclasts differentiation (Masuyama et al., 2008). TRPV4 is known as a sensor of mechanical or osmotic signals (Mizuno et al., 2003; Suzuki et al., 2003). TRPV4-mediated Ca^2+^ influx appeared necessary for sustained Ca^2+^ signaling, NFATc1 gene transcription, terminal differentiation and osteoclast activity (Masuyama et al., 2008, 2012). TRPV1 activation was also observed to cause osteoclasts, *in vivo* bone loss. It has also shown a weak response to low pH (Suzuki et al., 2003), and got activated by src under acidic conditions. Taken together, this data suggests that extracellular acidosis is activated by TRPV4. Its antagonist, RN1734, partially inhibited acidosis-induced osteoclast formation, while its agonist 4-α PDD enhanced osteoclast formation under mild acidosis, thus implicating TRPV4 in acid-induced osteoclast formation (Kato and Morita, 2013). Moreover, there is also a possibility that acidosis-induced osteoclast formation is regulated by other unidentified TRP family cation channels permeable to Ca^2+^. Consistent with this probability, experiment by Kato and Morita (2013) have already shown that Ruthenium red, a general blocker of TRP channels, potently inhibited the acidosis-induced osteoclast formation.

### ASICs

**Figure 2:**
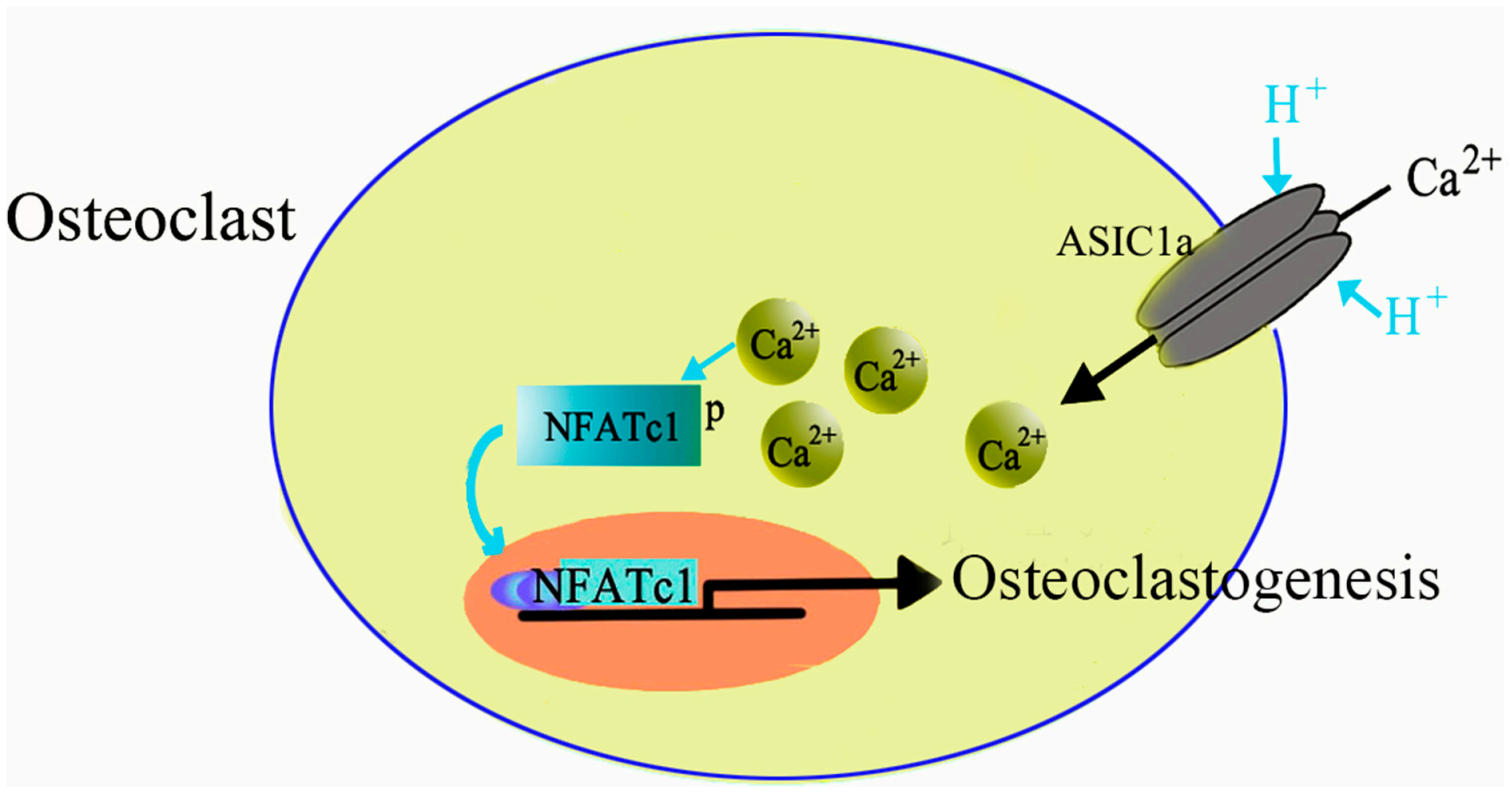
**Role of ASIC1a in osteoclasto genesis**.

ASICs are proton-gated channels that are distributed throughout the central and peripheral nervous systems. Currently, there are six isoforms of ASICs (Xiong et al., 2004; Yuan et al., 2010b) and are activated by a decrease in the extracellular pH and are cation-selective. Recently it has been suggested that ASICs are expressed not only in nervous system but also in the non-neuronal cells, such as dendritic cells (Tong et al., 2011), astrocytes (Huang et al., 2010), vascular smooth muscle cells (Grifoni et al., 2008; Jernigan et al., 2012), nucleus pulposus cells (Ohtori et al., 2006; Uchiyama et al., 2007, 2008; Navone et al., 2012; Cuesta et al., 2014; Sun et al., 2014), synoviocytes (Kolker et al., 2010), hepatic stellate cells (Wu et al., 2014), and glioma cells (Berdiev et al., 2003; Vila-Carriles et al., 2007; Kapoor et al., 2009). The wide expression pattern suggests that ASICs may have more diverse role in physiological and pathogenic processes. We, and others, have recently reported the expression of ASICs in human skeleton and rat articular and endplate chondrocytes (Jahr et al., 2005; Yuan et al., 2010a,b; Hu et al., 2012; Rong et al., 2012). Moreover, our study also showed the involvement of ASICs in inducing osteoclast differentiation in response to acidosis (Li et al., 2013). The mRNA of four ASICs subtypes, including ASIC1a, ASIC1b, ASIC2a, and ASIC3 is expressed in the osteoclasts derived from RANKL and M-CSF induced BMMs. The acidosis has been reported to increase the mRNA expression of ASIC1a in osteoclasts, suggesting that ASIC1a has a role in facilitating the sensing and response to differences in extracellular pH. Furthermore, we identified that ASIC1a is essential for the extracellular acidification-induced increase in [Ca^2+^]i levels in osteoclasts and also involved in extracellular acidification-stimulated NFATc1 signaling in osteoclastogenesis (Figure 2). Taken together, our data suggested that ASIC1a seems to be involved in acid-induced osteoclast differentiation and bone resorption and can act as a potential therapeutic target for the treatment of human diseases, such as osteoporosis (Li et al., 2013).

## Conclusion

Recent evidences based on animal model studies, implicate several acid sensing receptors in various diseases. However, going forward it will be important to determine how our current knowledge of acid sensing receptors can be translated to human patients. One way would be to first identify if there are any existing genetic associations between acid sensing receptors and human bone resorption-related diseases. Another path would be to directly test if inhibitor of acid sensing receptors can produce beneficial effects in human patients. In addition, a better understanding of the molecular mechanisms involved in action of acid sensing receptors will help to clarify how inhibiting or potentiating these receptors could affect the pathophysiology and behavior of human patients. In this review, we have tried to highlight the critical role of acidosis in osteoclast fusion, differentiation, activity, survival, adhesion, migration and acid sensing mechanism. This effort will help to provide further insight into the molecular and cellular understanding of bone resorptive disorders in an acidic environment.

## Author contributions

All authors wrote, edited and approved the final submission of the manuscript. FY, JD, HX, and WF conceived the project and designed experiments. XL, MX, HX, and JD performed experiments and collected and analyzed data. All authors developed analytical tools.

### Conflict of interest statement

The authors declare that the research was conducted in the absence of any commercial or financial relationships that could be construed as a potential conflict of interest.
